# Pelvic insufficiency fracture after definitive radiotherapy for uterine cervical cancer: retrospective analysis of risk factors

**DOI:** 10.1093/jrr/rrt055

**Published:** 2013-05-17

**Authors:** Haruka Uezono, Kayoko Tsujino, Keno Moriki, Fumiko Nagano, Yosuke Ota, Ryohei Sasaki, Toshinori Soejima

**Affiliations:** 1 Department of Radiation Oncology Hyogo Cancer Center, 13–70 Kitaoji-Cho, Akashi, Hyogo 673-8558, Japan; 2 Department of Diagnostic Radiology, Hyogo Cancer Center, 13–70 Kitaoji-Cho, Akashi, Hyogo 673-8558, Japan; 3 Division of Radiation Oncology, Kobe University Graduate School of Medicine, 7-5-2 Kusunoki-Cho, Chuou-Ku, Kobe, Hyogo 650-0017, Japan

**Keywords:** adverse event, CT density, pelvic insufficiency fracture, radiotherapy, uterine cervical cancer

## Abstract

The purpose of this study is to determine the incidence, clinical characteristics and risk factors of postradiation pelvic insufficiency fracture (PIF) in women with uterine cervical cancer. We reviewed the medical records of 126 patients who received definitive radiotherapy (RT) for uterine cervical cancer between 2003 and 2009 at our institution. Among them, 99 patients who underwent at least one computed tomography (CT) or magnetic resonance imaging of the pelvis during their follow-up at more than 6 months were included in this analysis. The relationship between the incidence of PIF and several patient- and treatment-related factors was analyzed. The median follow-up period was 21 months. Of the 126 patients, 33 (with a total of 50 lesions) were diagnosed with PIF. The 2-year cumulative incidence was 32%. Univariate analysis showed that age ≥70 years (*P*= 0.0010), postmenopausal state (*P* = 0.0013), and lower CT density of bone and bone marrow (*P*= 0.020) significantly related to PIF. In a multivariate analysis, of the 59 patients whose CT densities were evaluable, lower CT density was the only significant factor associated with PIF (*P* = 0.0026). In conclusion, postradiation PIFs were detected in a considerable number of patients after definitive RT for cervical cancer. Predisposing factors were older age, postmenopausal state, and decreased density of bone and bone marrow on CT.

## INTRODUCTION

Radiotherapy (RT) has been considered an essential method for the treatment of uterine cervical cancer. Survivorship has improved since the introduction of platinum-based chemoradiotherapy, and the late effects of the treatment have drawn more attention. Although postradiation pelvic insufficiency fracture (PIF) was considered a relatively rare adverse event after pelvic irradiation, several clinical investigations have shown that postradiation PIF is more common than previously thought [1–8]. Injury and occlusion of the microvasculature of mature bone and stasis of osteoclasts and osteoblasts are thought to be major mechanisms of RT-induced insufficiency fracture [9]. Recent studies suggest that older age, postmenopausal status, lower body weight or body mass index (BMI), RT intensity (the larger volume and dose irradiated, and the use of concurrent chemotherapy), and higher numbers of deliveries are risk factors for RT-induced PIF [1–3, 7]. In the general population, postmenopausal state, high-dose corticosteroid use, rheumatoid arthritis, diabetes mellitus, heparin use, low body weight, and smoking history are known to be associated with pelvic insufficiency fracture [10].

To our knowledge, no studies have addressed the correlation between postradiation insufficiency fracture and the quantitative assessment of osteoporosis. We retrospectively evaluate the incidence of PIF at our institution and analyze the associated risk factors, including the quantitative assessment of osteoporosis.

## MATERIALS AND METHODS

### Study population

The RT records and the medical charts of 126 patients with cervical cancer who received definitive RT consisting of external beam (EBRT) and high-dose-rate intracavitary brachytherapy (HDR-ICBT) at our institution between January 2003 and December 2009 were reviewed. We obtained written consents from all patients in this study regarding the use of their medical records for the purpose of clinical studies. Patients who received postoperative adjuvant RT were not included. Of the 126 patients, 99 who underwent at least one pelvic computed tomography (CT) or magnetic resonance imaging (MRI) during their follow-up at > 6 months after the initiation of RT were included in this study. Pretreatment CT images for RT planning were available for all patients for comparison. Patient characteristics are described in Table 1.

**Table 1.** Patient characteristics

Characteristics	
Median age (range)	68 (31–95) years
Median BMI (range)	21.4 (15.8–32.8)
Menopausal status	
premenopausal	14
postmenopausal	85
Smoking status	
current/past	21
never	73
unknown	5
Past medical history	
diabetes mellitus	14
rheumatoid arthritis	5
Stage (FIGO)	
IB	5
IIA	3
IIB	26
IIIA	4
IIIB	47
IVA	14
Histopathology	
squamous cell carcinoma	86
adenocarcinoma	7
adenosquamous cell carcinoma	2
poorly differentiated carcinoma	2
neuroendocrine and squamous cell carcinoma	1
unknown	1

FIGO = International Federation of Gynecology and Obstetrics.

### Treatment

All patients received both EBRT and HDR-ICBT. EBRT was delivered to the whole pelvic (WP) field followed by the same WP field with central shielding. EBRT was prescribed with a median prescribed dose of 50.4 Gy (range, 45.0–50.4 Gy) at 1.8 Gy per fraction, five days per week, using 10-MV photons. A 4-field box technique was used in 78 patients (79%) and an anteroposterior/posteroanterior (AP/PA) field was used in 21 patients (21%). In general, we used a 4-field box technique unless the patient had undergone hip-replacement surgery, had severe osteoarthritis in the hip joint, required irradiation to the inguinal region, or had a thin body shape in which the distance between the anterior and posterior abdominal wall was less than 20 cm. Central shielding was used in 97 patients after the WP RT. The median prescribed dose for central shielding field was 14.4 Gy (range, 5.4–34.2 Gy). Boost EBRT was added to metastatic lymph nodes in 19 cases with 4–8 Gy (median 6 Gy) at 1.8–2.0 Gy per fraction. All EBRT was planned with the CT-based treatment-planning system (FOCUS 9200 ver. 3.2.1, XiO ver. 4.20 – ver.4.60, CMS/Elekta, Stockholm, Sweden).

WP fields were designed as described below. The superior border was set at either the L4–5 intervertebral level or the aortic bifurcation, depending on which was located more superiorly. In patients with positive para-aortic nodes, the RT fields were extended up to levels in which the involved lymph nodes were adequately included. The inferior border was at the bottom (inferior margin) of the obturator foramen, or 2–3 cm below the lowest extent of the cervical or vaginal involvement. The lateral border was at 1.5–2 cm lateral to the external margin of the small pelvis. The anterior border was at 0–1 cm anterior to the pubic symphysis, and the posterior border was adjusted to the posterior edge of the sacrum. Every field was confirmed to include the lymphatic area contoured on CT.

HDR-ICBT was delivered to all patients with the Fletcher-Williamson applicator (tandem and ovoid) system or tandem and segmented cylinder applicator. We used a microSelectron HDR and PLATO (Nucletron/Elekta) as a radioactive source delivery and a treatment-planning system, respectively. The median total dose of HDR-ICBT was 22 Gy (range, 6–30 Gy), 5–6 Gy per fraction to point A, weekly. The median total dose prescribed to point A (WP EBRT and HDR-ICBT) normalized to biological effective dose (BED) at α/β = 3 and α/β = 10 were 120.2 Gy (range, 84.2–139.0 Gy) and 74.5 Gy (range, 36.3–84.1 Gy), respectively.

Chemotherapy was administered to 68 patients (concurrently and sequentially in 67 patients and 1 patient, respectively). Therapeutic regimens of concurrent chemotherapy consisted of weekly nedaplatin (cis-diammine-glycoplatinum) at 30 mg/m^2^ (58 of 67, 87%), weekly cisplatin at 30–40 mg/m^2^ (7 of 67, 10%), and irinotecan at 40 mg/m^2^ (2 of 67, 3%). Nedaplatin, a derivative of cisplatin with similar antitumor activity but less renal and gastrointestinal toxicity, is commonly used in Japan. Neoadjuvant and adjuvant chemotherapy were administered in eight and three patients, respectively.

### Follow-up and diagnostic criteria of PIF

For follow-up, patients visits were scheduled at least once a month until 6 months, every 2 months from 6–12 months, every 3 months from 1–5 years, then once a year until 10 years after completion of the treatment. As to a follow-up imaging study, abdominal CT and/or pelvic MRI were performed once at 1–3 months initially, then every 6 months until two years, and, generally, once a year after that. We also planned additional imaging study as needed at times of suspicion of recurrent disease or patient symptoms. Post-treatment CTs/MRIs were performed a median of 3 times (range, 1–14 times) during the median clinical follow-up period of 25 months (range, 9–89 months). The median time from the start of RT to the latest imaging study was 21 months (range, 6–85 months).

All imaging studies were reviewed separately by a diagnostic radiologist and a radiation oncologist (KM and HU). We defined the diagnostic criteria of insufficiency fracture as a bony lesion with an apparent fracture line, with or without sclerotic change detected by CT and/or MRI, within the irradiated field. Traumatic or metastatic lesions were excluded by history, clinical course, and radiological appearance. A bone marrow change of only sclerosis or edema without cortical disruption was not defined as insufficiency fracture. Osteonecrosis of the hip was counted as insufficiency fracture. Fracture sites were categorized into six subgroups including the spinal column, sacrum, sacroiliac joint, ilium, pubis, and femoral head. Some cases had multiple fracture lines, mainly on the sacrum and one or two fracture lines involving the sacroiliac joint; those cases were categorized as sacral fracture. Discordant image interpretations for the two readers were solved by discussion and careful reviewing. We used a uniform method for all patients, diagnosing PIF only when a fracture line was confirmed on CT and/or MRI. A fracture line showed as a sharp, linear, low-density area on CT, and a low-signal-intensity area on a T1-weighted image.

### Measurement of bone and bone marrow CT density

**Fig. 1.**
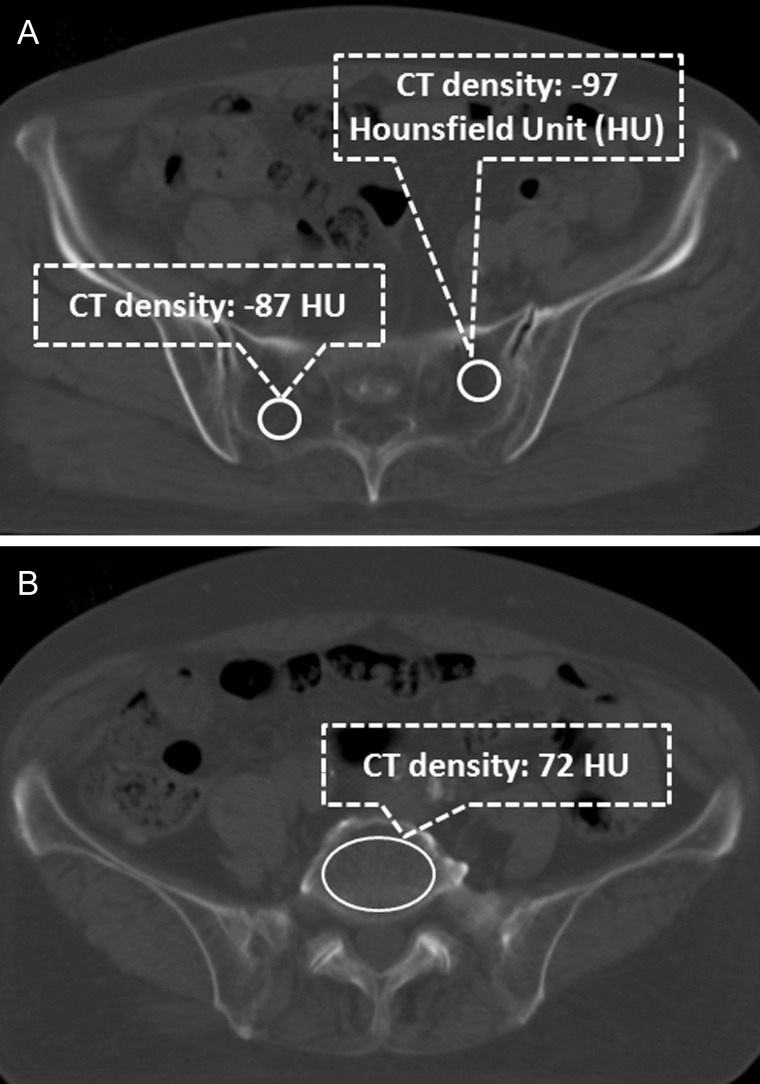
(**A**) Transaxial image of an RT-planning CT of a representative case. Area of visually lowest density of bone marrow in the right and left sacrum coincided with each other in this case. CT densitometry shows −87 and −97 HU on the right and left sacral bone marrow, respectively. (**B**) Transaxial image of a RT-planning CT of the same case as Fig. 1A. L5 lumbar vertebra shows the most homogenous bone marrow density on this slice. CT densitometry shows 72 HU on L5 lumbar vertebra.

We measured the pretreatment CT density (in Hounsfield Unit [HU]) of 59 consecutive patients as obtained through a specific imaging viewer (Synapse, FUJIFILM Medical Co., Ltd, Tokyo, Japan). The 59 patients' imaging data that is acquired in 2006 and thereafter were obtainable through the imaging viewer (Synapse) with the function of CT densitometry. However the other 40 patients' imaging data, acquired before 2006, were not obtainable through the imaging viewer. To reduce mis-estimation, we selected seven different sites of bone marrow within the general irradiation field. We selected three different transaxial images showing visually the lowest bone-marrow density on bone windows in the right and left side of the sacrum. A 1-cm diameter circle region of interest was utilized to measure the CT density of bone and bone marrow on each of the six slices. We calculated the mean value for the three on each side. We selected the transaxial image that appeared the most homogenous for the L5 vertebra, and measured the vertebral bone and bone-marrow density. Each of the three values (L5 vertebra, right and left sacrum) and the mean of the three values were used for analysis. A representative case is shown on Fig 1.

### Dose-volume analysis

Dosimetric analyses of EBRT were performed on 70 consecutive patients whose EBRT planning data were available. We contoured eight segments of pelvic bone including the fifth lumbar vertebra, sacrum, right and left sacroiliac joints, ilium and pubis on the treatment-planning CT. Dosimetric parameters including V20, 30, 40, 50 and the mean dose of each segment were calculated, and the relationship to the incidence of PIF was evaluated.

### Endpoints and statistical data analysis

We defined the onset of PIF as the date of detection on imaging study. In patients with multiple injuries detected separately, the date of the first event onset was recorded. The date of the latest imaging study was counted for censoring. The cumulative incidence rate of PIF was calculated by the Kaplan-Meier method. The relationship between the incidence of PIF and the potential risk factors (age, menopausal status, type II diabetes mellitus, rheumatoid arthritis, RT technique, RT dose, and chemotherapy use) were analyzed by the log-rank test (univariate analysis). Multivariate analysis using the Cox proportional hazard model was carried out on those factors with *P* < 0.1 in the univariate analysis. The patient population whose CT density was not available was excluded from multivariate analysis. *P*-values less than 0.05 were considered statistically significant. Correlation between CT density of bone and bone marrow, and postmenopausal status and age were analyzed by *t*-test. We also analyzed the relationship between age and disease stage, and chemotherapy use by *t*-test. Statistical data analyses were performed using JMP 8 (SAS institute, Cary, NC, USA).

## RESULTS

**Fig. 2.**
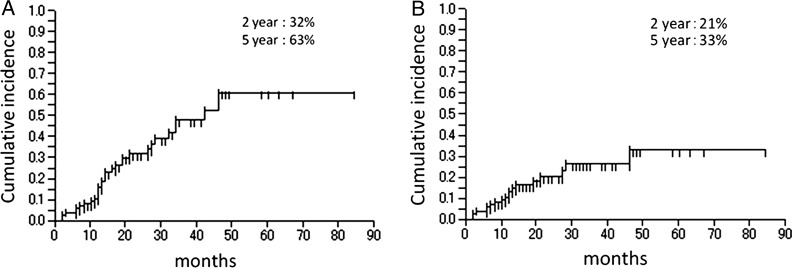
(**A**) Cumulative incidence of overall PIF after pelvic radiotherapy for uterine cervical cancer. The 2- and 5-year cumulative incidences of overall PIF were 32% and 63%, respectively. (**B**) Cumulative incidence of symptomatic PIF. The 2- and 5-year cumulative incidences were 21% and 33%, respectively.

**Fig. 3.**
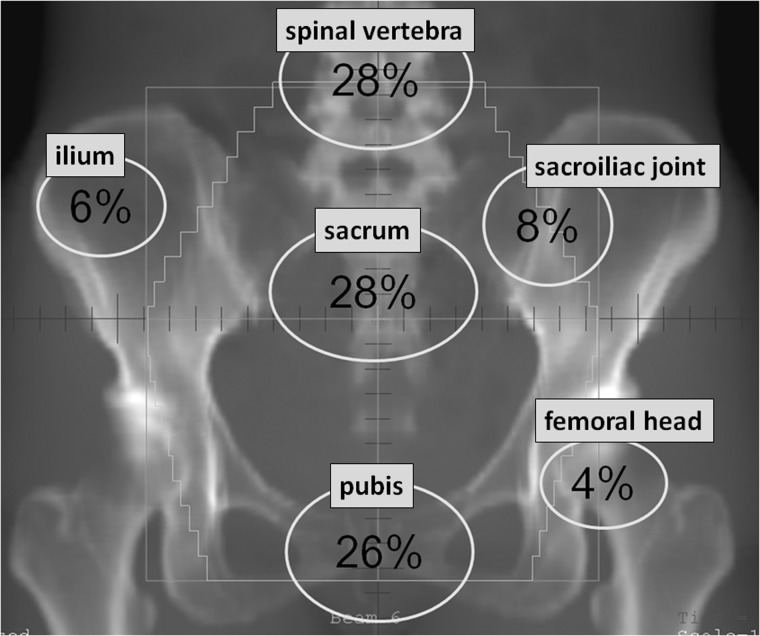
Distribution and proportion of PIF in a general anteroposterior/posteroanterior pelvic field after radiotherapy for uterine cervical cancer. Fracture lines and bone necrosis within the irradiated field were counted as PIF.

Of the 99 patients, 33 (with 50 lesions) were diagnosed with PIF within the irradiated field. The time interval between RT initiation and PIF detection was 2–46 months (median, 14 months). The 2- and 5-year cumulative incidences of overall PIF were 32% and 63%, respectively (Fig. 2A). Lumbar or pelvic pain which was considered to be related to PIF developed in 20 patients (61%). Among these, 16 patients achieved pain relief with nonsteroidal anti-inflammatory drugs, but three required narcotic analgesics. One patient with femoral head necrosis underwent hip replacement surgery because of intolerable pain with conservative therapy. The onset of symptoms varied from 1–60 months (median, 11 months). The 2- and 5-year cumulative incidences of symptomatic PIF were 21% and 33%, respectively (Fig. 2B). The distribution of PIF in the patients was as follows: lumbar spinal vertebra in 14 (28%), sacrum in 14 (28%), pubis in 13 (26%), sacroiliac joint in 4 (8%), ilium in 3 (6%), and femoral head in 2 (4%) (Fig. 3). Most pubic fractures were seen adjacent to the symphysis pubis.

The relationship between the occurrence of PIF and clinical- and treatment-related factors are summarized in Table 2A. Univariate analysis revealed age ≥70 years (2-year incidence 47% vs 16%, *P* = 0.0010), postmenopausal status (2-year incidence 36% vs 0%, *P* = 0.0013), and mean CT density < 35 HU (2-year incidence 46% vs 24%, *P* = 0.020) as statistically significant predisposing factors for developing PIF. CT density measured at the three sites independently showed the relationship with PIF (Table 2B). Patients who did not receive chemotherapy showed a higher incidence of PIF than those who did. There were significant correlations between CT density, postmenopausal status and age. The mean CT density of pre- and postmenopausal patients was 37 and 105 HU, respectively (*P* = 0.0008). The mean age of CT density ≤ 35 HU and > 35 HU were 75 and 60 years, respectively (*P*≤ 0.0001). Younger patients were prone to more advanced disease in this study. Comparing Stage I–II with III–IV, the mean age of each group was 70 and 65 years, respectively (*P*= 0.09). In addition, younger patients were more likely to receive chemotherapy. The mean age of patients who received chemotherapy and who did not receive was 61 and 77 years, respectively (*P* < 0.0001). Although some factors may be confounding in part, multivariate analysis revealed that lower CT density was the only significant predisposing risk factor (Table 3). As the CT densities were evaluable only in 59 of the 99 cases, we performed multivariate analysis on the whole cohort (*n* = 99) without the CT density factor. We found no significant difference for age (*P* = 0.07), menopausal state (*P* = 0.09) or the use of chemotherapy (*P* = 0.99).

**Table 2A.** Univariate analysis of risk factors associated with PIF (*n* = 99)

Factors		*P* = value	HR	95% CI
Age (years)	≥70	0.0010*	3.6	1.64–9.03
	<70			
BMI	≤21	0.94	1.03	0.49–2.10
	>21			
Menopausal status	Post	0.0013*	1550581	2.83–
	Pre			
Smoking history	Current/past	0.45	0.72	0.26–1.65
	never			
	unknown			
Diabetes mellitus	+	0.62	1.26	0.47–2.88
	−			
Rheumatoid arthritis	+	0.56	1.58	0.25–5.29
	−			
Chemotherapy use	+	0.057	0.51	0.25–1.02
	−			
EBRT dose (Gy)	≥50.4	0.48	0.72	0.32–1.93
	<50.4			
Number of ports	4	0.70	0.85	0.39–2.12
	2			
RT dose^a^ (α/β = 3)	≥120	0.43	0.78	0.38–1.54
	<120			
Mean CT density (HU)	<35	0.020*	3.09	1.18–9.60
	≥35			

CT density is listed separately because of a discrepancy in patient number.

**Table 2B.** Univariate analysis of CT density and PIF (*n* = 59)

Measured site	CT density (HU)	PIF + (*n* = 21)	PIF− (*n* = 38)	*P*-value
5th lumbar vertebra	≤130	17	14	0.013
	>130	4	24	
Right sacrum	≤−35	20	16	0.0033
	>−35	1	22	
Left sacrum	≤−35	19	19	0.027
	>−35	2	19	
Mean of the three sites	<35	16	12	0.020
	≥35	5	26	

HR = hazard ratio, CI = confidence interval. *P < 0.05. ^a^RT dose of whole pelvic EBRT and HDR-ICBT normalized to BED at point A.

**Table 3.** Multivariate analysis of risk factors associated with PIF (*n* = 59)

Factors	*P*-value	HR	95% CI
Age (years)	0.31	8.82	0.14*–*589
Menopause (pre/post)	0.38	191127^a^	0*–*
Chemotherapy (+/−)	0.40	0.60	0.18*–*1.95
Mean CT density (HU)	0.0026*	203	5.84–11582

HR = hazard ratio, CI = confidence interval. *P < 0.05. ^a^No event was found in the premenopausal group.

**Fig. 4.**
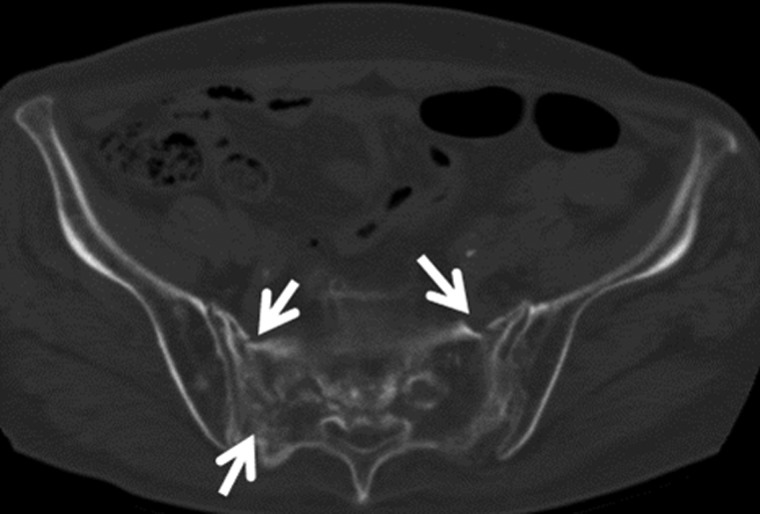
A follow-up CT image obtained 12 months after treatment shows multiple sacral fracture lines (arrows).

CT density obtained from L5 and the right and left sacrum ranged from 25 to 302, −108 to 55, and −110 to 53 (median, 129, −49, and − 55) HU, respectively. The mean of the three values ranged from −42.5 to 177.5 (median, 35.5) HU. A representative case of sacral PIF is shown in Fig. 4. An 80 year-old woman with International Federation of Gynecology and Obstetrics (FIGO) stage IIIB uterine cervical cancer (the same case as presented in Fig. 1) treated with definitive RT developed pelvic pain 12 months after the start of RT. A transaxial image of follow-up CT shows multiple fracture lines in the sacrum (Fig. 4).

We performed dosimetric analyses of EBRT on 70 patients (Table 4). Although not statistically significant, a lower dose of EBRT showed a higher fracture rate trend in the left pubis. The 2-year incidence of PIF in groups with EBRT doses of >43 Gy and ≤43 Gy were 7% vs 14%, respectively (*p* = 0.26). On the other hand, patients who received a higher total dose of HDR-ICBT were prone to left pubic insufficiency fracture in the group of 70 patients in which EBRT dose-volume correlation was analyzed. The 2-year incidences of PIF in the groups of HDR-ICBT dose >22 Gy and ≤22 Gy were 16% vs 7%, respectively (*P* = 0.16). However, a statistically significant correlation was not observed between dose-volume histogram (DVH) parameters and the incidence of PIF.

**Table 4:** Dose-volume association with PIF

site	index	PIF +	PIF −	*P-*value
		Mean ± SE	Mean ± SE	
Right pubis	*n*	7	63	
	Mean (cGy)	4202.4 ± 133.0	4252.1 ± 44.3	0.64
	V50 (%)	32.3 ± 6.4	29.3 ± 2.2	0.33
	V40 (%)	64.0 ± 5.0	68.7 ± 1.7	0.81
	V30 (%)	86.0 ± 3.7	87.7 ± 1.2	0.66
	V20 (%)	96.7 ± 1.8	96.9 ± 0.6	0.54
Left pubis	*n*	7	63	
	Mean (cGy)	4121.4 ± 125.7	4293.4 ± 41.9	0.90
	V50 (%)	20.00 ± 6.7	31.7 ± 2.2	0.95
	V40 (%)	64.0 ± 5.0	69.9 ± 1.7	0.87
	V30 (%)	85.0 ± 3.2	88.5 ± 1.1	0.85
	V20 (%)	95.7 ± 1.2	97.4 ± 0.4	0.89
Sacrum	*n*	9	61	
	Mean (cGy)	4585.0 ± 98.0	4599.3 ± 37.7	0.89
	V50 (%)	43.9 ± 6.9	40.1 ± 2.7	0.30
	V40 (%)	81.2 ± 3.7	82.7 ± 1.4	0.64
	V30 (%)	95.4 ± 1.6	96.8 ± 0.6	0.45
	V20 (%)	99.9 ± 0.6	99.6 ± 0.2	0.31
L5	*n*	11	59	
	Mean (cGy)	4226.6 ± 264.4	4045.1 ± 114.1	0.27
	V50 (%)	14.9 ± 5.3	19.2 ± 2.3	0.77
	V40 (%)	58.0 ± 7.8	59.2 ± 3.4	0.89
	V30 (%)	96.6 ± 6.9	88.8 ± 3.0	0.15
	V20 (%)	99.5 ± 5.8	92.5 ± 2.5	0.14

SE = standard error.

## DISCUSSION

The 2-year cumulative incidence rate of PIF after radiotherapy was 32% in the current study. Estimated predisposing risk factors were older age, postmenopausal status, and decreased CT density of bone and bone marrow. The novel observation of this study is that pretreatment CT density is associated with the development of PIF.

In our series, 33 of 99 patients (33%) developed PIF after definitive RT for uterine cervical cancer. Recent studies have revealed that the postradiation PIF rate is 7–34% [2–8]. The reported cumulative incidences at two and five years were 10–37% and 8–45%, respectively [1–7]. The current study showed a relatively higher rate than previous reports, which we believe is partly because the current study was restricted to patients who underwent curative RT with HDR-ICBT. We evaluated both symptomatic and asymptomatic patients, even though a previous study [11] evaluated only symptomatic patients. Furthermore, our study population was relatively older than previous reports [2–5]. The median time interval between the first day of RT and detection of PIF was 14 months in our study, which is almost equivalent to previous studies (6–16.9 months) [2–5, 7, 11].

Based on our analysis, a lower CT density of bone and bone marrow is a significant predisposing factor for developing postradiation PIF. Although quantitative CT is known to reflect bone mineral density (BMD) [12], limited evidence has shown the efficacy of conventional CT density for this purpose. Nishihara *et al*. [13] showed that the CT density obtained from the vertebral body can be used for the estimation of bone mineral content. Link *et al*. [14] revealed a close relationship between the CT density obtained from spiral CT and quantitative CT. We believe our results indicate and support the close relationship between osteoporosis and postradiation PIF that has been noted by some investigators [15]. BMD evaluation is not a routine work-up modality in cervical cancer management. In our series, BMD data were available for very few patients. Based on our data and previous studies [13, 14], the conventional CT density obtained for RT planning may have the potential for predicting the occurrence of subsequent PIF. Osteoporosis screening at initial presentation and appropriate medical intervention may contribute to the reduction of PIF and improve the patient's quality of life. Bisphosphonates have been shown to reduce cancer-induced bone loss effectively and can be a treatment choice or prophylaxis for postradiation PIF. In fact, bisphosphonate use is advocated by some investigators [3, 11]. However, we are cautious about introducing bisphosphonate use for postradiation PIF prophylaxis because radiation and bisphosphonates have the same antiangiogenic effects, which decrease bone turnover [9, 16]. The combined use of bisphosphonates and radiation may therefore increase the risk of osteonecrosis. Further study is warranted to confirm the tolerability and efficacy of this approach.

Our study has several limitations. First, this was a single-institution, retrospective study. The medical records, including patients' symptoms and prescribed medications, may not be fully accurate. The interval and protocol of imaging studies were not standardized, causing a detection bias. Post-treatment MRIs were targeted mainly to the primary disease, which may have missed fractures of the superior pelvis and spine. Second, the follow-up period was not long enough to estimate the true incidence of fracture rate accurately. Third, the evidence correlating CT density and bone mineral density was limited. Although there are some limitations, this study is the first report showing that the pretreatment CT density of bone and bone marrow is significantly related to subsequent PIF in patients who have undergone definitive RT for uterine cervical cancer. In addition, the current study was designed to analyze uniformly treated patients of uterine cervical cancer who had undergone both EBRT and ICBT.

Although the sacrum and sacroiliac joint are known to be the most commonly injured, we have reported a relatively low rate in the sacroiliac joint. This is because we categorized as sacral fracture, not sacroiliac fracture, so long as the fracture line involvement to the sacroiliac joint was minimal. It is difficult to clearly differentiate between sacral fracture and sacroiliac joint fracture on imaging study. Some previous studies do not refer to the bone complication in the spine. So the incidence of sacral and sacroiliac fracture appears to be relatively low in this study compared with some previous reports. Ikushima *et al*. [5] showed that fifth lumbar spinal fracture occurred in 22% of the cases. The incidence of lumbar spinal vertebral fracture does not appear to be particularly high in the current study.

Concurrent platinum-based chemotherapy is thought to be essential in cervical cancer therapy [17]. Although this is known to increase the toxicity of RT [18], we did not find evidence that this increased the incidence of fracture. On the contrary, patients who did not receive chemotherapy tended to have an increased fracture rate. Likewise, a lower total dose (WP EBRT plus HDR-ICBT, normalized to BED [α/β = 3]) was associated with a higher incidence of fracture in the 99 patients. We assumed that this negative correlation occurred because a more advanced stage of the disease requiring more intensive treatment tended to occur in younger patients. Because we used different treatment-planning systems for EBRT and HDR-ICBT, it was impossible to calculate the total dose distribution combining EBRT and HDR-ICBT. The contribution of the EBRT dose to PIF was low in this study, which we presume is partly because the EBRT field did not vary much on a case-by-case basis in uterine cervical cancer treatment. Although it was not evident in our study, we assume that there still might be a correlation between irradiated dose, volume and fracture rate [19]. Rubin noted that the risk of radiation damage to an organ is generally proportional to the dose and volume irradiated. IMRT will be a promising option for dose reduction to bones as it is studied as an option for lessening hematologic toxicity [20, 21]. Further studies are required to confirm this assumption.

The dose-volume analysis in the current study suggests that the HDR-ICBT dose contributes to pubic fracture, although this was not statistically significant. Tai *et al*. and Fu *et al*. reported that 5–20% of prescribed brachytherapy doses affect the pubic bone [11, 22]. Analyzing a patient population with postoperative RT who have not received HDR-ICBT may provide the additional data required to ascertain the effect of HDR-ICBT.

## CONCLUSION

In conclusion, PIF after definitive RT for uterine cervical cancer developed in 33% of patients. The estimated 2- and 5-year prevalence rates were 32% and 63%, respectively. Statistical analysis revealed that older age, postmenopausal state, and decreased CT density of bone and bone marrow were the predisposing risk factors.
